# Enhancing high-throughput optogenetics: Integration of LITOS with Lustro enables simultaneous light stimulation and shaking

**DOI:** 10.17912/micropub.biology.001073

**Published:** 2024-02-02

**Authors:** Zachary P Harmer, Thomas C Höhener, Alex E Landolt, Claire Mitchell, Megan McClean

**Affiliations:** 1 Department of Biomedical Engineering, University of Wisconsin-Madison; 2 Institut für Zellbiologie, Universität Bern

## Abstract

Optogenetics is a powerful tool that uses light to control cellular behavior. Here we enhance high-throughput characterization of optogenetic experiments through the integration of the LED Illumination Tool for Optogenetic Stimulation (LITOS) with the previously published automated platform Lustro. Lustro enables efficient high-throughput screening and characterization of optogenetic systems. The initial iteration of Lustro used the optoPlate illumination device for light induction, with the robot periodically moving the plate over to a shaking device to resuspend cell cultures. Here, we designed a 3D-printed adaptor, rendering LITOS compatible with the BioShake 3000-T ELM used in Lustro. This novel setup allows for concurrent light stimulation and culture agitation, streamlining experiments. Our study demonstrates comparable growth rates between constant and intermittent shaking of *Saccharomyces cerevisiae* liquid cultures. While the light intensity of the LITOS is not as bright as the optoPlate used in the previous iteration of Lustro, the constant shaking increased the maturation rate of the mScarlet-I fluorescent reporter used. Only a marginal increase in temperature was observed when using the modified LITOS equipped with the 3D-printed adaptor. Our findings show that the integration of LITOS onto a plate shaker allows for constant culture shaking and illumination compatible with laboratory automation platforms, such as Lustro.

**Figure 1. LITOS adapted for constant shaking:**
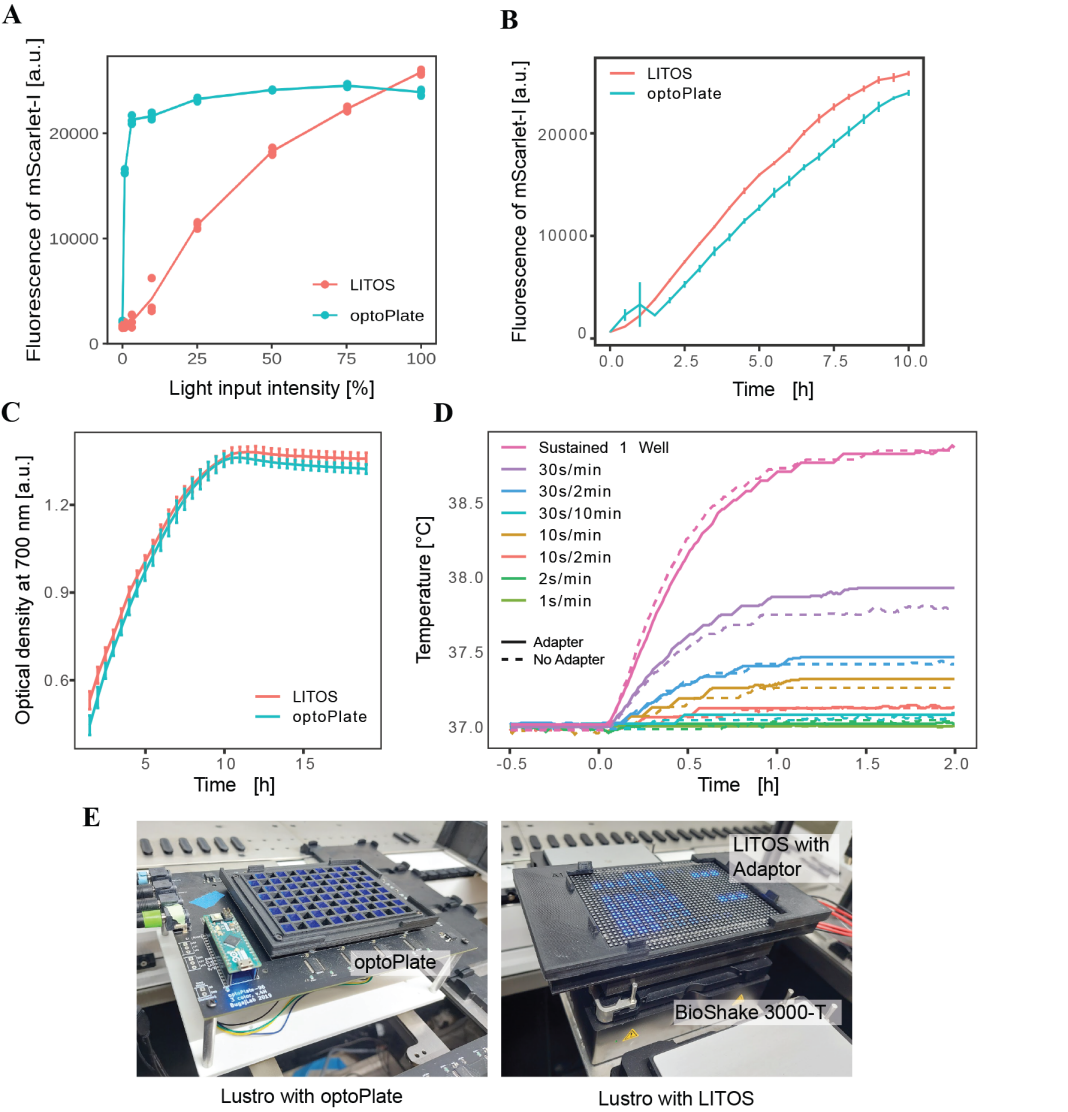
A) A strain with a CRY2PHR/CIB1 optogenetic split transcription factor driving expression of mScarlet-I (yMM1731) is induced on the optoPlate (with intermittent shaking) or on the LITOS (with constant shaking) under a range of programmed light intensities (in 200 µL volumes). 100% intensity corresponds to 1500 µW/cm ^2^ on the optoPlate and 20 µW/cm ^2^ on the LITOS. Fluorescence shown was measured at 10 hours into induction. B) Fluorescence over time for the maximum light intensity conditions of the strain on the LITOS (constant shaking) and optoPlate (intermittent shaking). C) Optical densities of a strain grown with intermittent shaking on the optoPlate or constant shaking on the LITOS mounted on the BioShake 3000-T ELM. D) Temperature of the LITOS with the adapter (solid lines) and without the adapter (dotted lines) for different light pulsing conditions over two hours. E) Photos of the Lustro setup using either the optoPlate or LITOS (mounted on the BioShake shaker).

## Description

**Introduction:**

Optogenetics has transformed the field of cellular biology by using light-sensitive proteins to precisely control cellular processes. The light-induced conformational change of the optogenetic protein can alter the protein’s binding activity or localization, which can be used to control gene expression, signaling pathways, and other biological behavior. In our previous work, we integrated optogenetics in an automation platform to make the Lustro platform (Harmer & McClean, 2023b, 2023a) . Performing optogenetic experiments with automation platforms like Lustro has the potential to significantly accelerate experimental workflows, providing researchers with a powerful tool for dynamic gene expression control.

Lustro comprises an illumination device, a shaking device, and a plate reader, all integrated with a Tecan Fluent Automation Workstation. The platform's robotic gripper arm facilitates the transfer of microwell plates between these components, following a programmed schedule. Lustro uses the optoPlate, designed by Bugaj and colleagues (Bugaj & Lim, 2019) for powerful illumination of cell cultures in microwell plates. Cell cultures are placed in the microplate, and after a period of light induction through individually programmable LEDs (using the optoPlate), the plate is moved to the shaking device to resuspend the cultures. However, due to the size and weight of the optoPlate, the shaking step must be performed separately because it exceeds the maximum capacity of the BioShakes. The plate is then moved to the plate reader to measure optical density and fluorescence, enabling frequent and reliable data collection. This automated process greatly expedites the prototyping and testing of optogenetic systems.

In this work, we substituted the optoPlate LED device with LITOS, an illumination system featuring an individually programmable LED matrix, rendering it compatible with multiple microplate formats (Höhener et al., 2022) . Thanks to its low cost, and ease-of-use, LITOS is a versatile tool for various optogenetics applications. Its lightweight and compact design permits seamless adaptation to cell culture shaking devices, facilitating concurrent illumination and agitation, thereby simplifying and optimizing the experimental process.

Adapting the LITOS to securely fit on the shaking device with a 3D-printed bottom adapter and top mask allows for simultaneous light stimulation and shaking. This reduces the amount of time the sample plate needs to be in the dark to take measurements, and allows light programs to be more continuously applied throughout an experiment (albeit at a lower light intensity). The constant shaking also improves fluorescent protein maturation rates, likely due to increased oxygenation rates (Hebisch et al., 2013) .

**Results:** We induced a strain with an optogenetic split transcription factor driving expression of a fluorescent protein (mScarlet-I). The optogenetic split transcription factor incorporates an optical dimerizer pair fused to a DNA-binding domain and an activation domain. The optical dimerizers bind each other upon blue light induction, reconstituting the split transcription factor and resulting in expression of the gene of interest. Induction was performed using either the optoPlate (with intermittent shaking) or the LITOS (adapted for use on the BioShake with constant shaking) with constant blue light illumination and different set intensities. The induction of the mScarlet-I reporter was measured every 30 minutes for 18 hours. A CRY2PHR/CIB1 split transcription factor strain (yMM1731) was selected, as it’s known to be sensitive to low light intensities( Figure 1A ) (Harmer & McClean, 2023b) . While the LITOS exhibited lower brightness compared to the optoPlate, it remained effective for inducing CRY2PHR/CIB1 and is compatible with the plate shaker. The sample induced on the LITOS (with constant shaking) increased in fluorescence more quickly than the sample on the optoPlate (with intermittent shaking), likely due to faster fluorescent protein maturation time with higher oxygen availability ( Figure 1B ) (Botman, et al. 2019; Guerra, et al., 2022; Hebisch et al., 2013) .

We then wanted to check whether the growth rate differs between illumination and shaking conditions. Therefore, we compared the growth rate of yeast cells grown with intermittent shaking (1 minute of shaking every 30 minutes) (Harmer & McClean, 2023b) to that of yeast cells shaken constantly on the LITOS with the BioShake, by measuring the optical density (OD) at 700 nm (to avoid bias from the red fluorescent reporter). Growth rates under constant and intermittent shaking were comparable, with slightly faster growth under constant agitation ( Figure 1C ).

Since we added 3D-printed adapters to the LITOS, we examined whether this would change the thermal equilibrium of the setup while it is running. Therefore, the temperature of samples on the device was measured over time, both with and without the mask. Temperatures in the modified LITOS with 3D-printed adapters were consistent with the original system, showing only marginal increases ( Figure 1D ).

**Discussion:**

The integration of LITOS with Lustro represents an important advancement in optogenetic research for cell types grown in suspension. This combined platform allows for concurrent light stimulation and culture agitation, streamlining experiments and providing valuable insights into cellular responses. Our findings suggest that both constant and intermittent shaking patterns are suitable for optogenetic studies. While LITOS may not match the brightness of the optoPlate, it remains effective for a range of optogenetic applications, particularly where performing optogenetic stimulation with shaking is desired in a laboratory automation context. The consistent temperature profiles in the modified LITOS with adapters demonstrate the robustness of this integrated system. Additionally, the versatile nature of this platform allows for further customization and integration with other experimental setups, opening doors to novel research avenues in dynamic gene expression control.

## Methods

**Integration of LITOS with Lustro:**

To attach LITOS to the BioShake 3000-T ELM shaker used in Lustro, 3D-printed adapters were designed (.stl files are in the Extended Data section or available on the LITOS GitHub page). The adapter consists of a top and a bottom part enclosing the LED matrix. This allowed it to be shaken vigorously enough to suspend yeast cells in liquid media (1000 rpm with 2 mm orbital), and to interface with the robotic gripper arm of the Fluent Automation Workstation. The shaking device clamps onto the base of the LITOS adapter to hold it in place. Fluorescence of mScarlet-I in the yeast cultures was read with a Tecan Spark plate reader. Light intensity was measured 1 cm above the bottom of the plate using a Thorlabs S120VC photodiode.

**Comparative Growth Analysis:**

Cultures were grown under constant and intermittent shaking conditions at room temperature (22°C). Shaking was performed at 1000 rpm with 2 mm orbital on a BioShake 3000-T ELM. Intermittent shaking was performed for 1 minute, repeated every 30 minutes. Optical densities (ODs) were measured at 700 nm (to avoid bias from the red mScarlet-I fluorescent protein) on a Tecan Spark plate reader to assess growth rates and the impact of agitation patterns. Strains used in this work were previously published (Harmer & McClean, 2023b) .

**Temperature Measurements:**

The effect of the 3D adapters on the heating of the medium in a well plate was determined by using an Arduino device equipped with multiple digital DS18B20 waterproof temperature sensors incubated at 37°C (as detailed in Höhener et al., 2022) . The LITOS systems with and without the attached adapters were measured in parallel.

**Materials Availability**

3D-printing files for the LITOS adapters can be found on GitHub: https://github.com/pertzlab/LITOS

## Extended Data

Description: STL file for LITOS bottom adapter. Resource Type: Model. DOI: 10.22002/sgaw9-y0e08

Description: STL file for LITOS top adapter. Resource Type: Model. DOI: 10.22002/15w7z-cwt37
